# Nuclease-resistant double-stranded DNA controls or standards for hepatitis B virus nucleic acid amplification assays

**DOI:** 10.1186/1743-422X-6-226

**Published:** 2009-12-22

**Authors:** Shuang Meng, Sien Zhan, Jinming Li

**Affiliations:** 1 Graduate School, Peking Union Medical College, Chinese Academy of Medical Sciences, PR China; 2 National Center for Clinical Laboratories, Beijing Hospital, Beijing, PR China

## Abstract

**Background:**

Identical blood samples tested using different kits can give markedly different hepatitis B virus (HBV) DNA levels, which can cause difficulty in the interpretation of viral load. A universal double-stranded DNA control or standard that can be used in all commercial HBV DNA nucleic acid amplification assay kits is urgently needed. By aligning all HBV genotypes (A-H), we found that the surface antigen gene and precore-core gene regions of HBV are the most conserved regions among the different HBV genotypes. We constructed a chimeric fragment by overlapping extension polymerase chain reaction and obtained a 1,349-bp HBV_C+S _fragment. We then packaged the fragment into lambda phages using a traditional lambda phage cloning procedure.

**Results:**

The obtained armored DNA was resistant to DNase I digestion and was stable, noninfectious to humans, and could be easily extracted using commercial kits. More importantly, the armored DNA may be used with all HBV DNA nucleic acid amplification assay kits.

**Conclusions:**

The lambda phage packaging system can be used as an excellent expression platform for armored DNA. The obtained armored DNA possessed all characteristics of an excellent positive control or standard. In addition, this armored DNA is likely to be appropriate for all commercial HBV DNA nucleic acid amplification detection kits. Thus, the constructed armored DNA can probably be used as a universal positive control or standard in HBV DNA assays.

## Background

Hepatitis B virus (HBV) infection is a major public health problem. It is responsible for chronic liver disease and is a risk factor for liver cirrhosis and hepatocellular carcinoma [1-3]. Early diagnosis and measurement of viral load in patients with HBV is very helpful for the management of this disease [2].

Real-time polymerase chain reaction (PCR) assays are widely used for the detection and quantification of HBV DNA in clinical samples [4-6]. Quantitative detection of HBV DNA in serum or plasma provides evidence about the level of viral replication, degree of infection, and efficacy of antiviral therapy [7-11]. Many commercial kits are available for HBV DNA quantification. Each kit uses proprietary HBV DNA standards or controls and provides results unique to that particular method. All of the HBV DNA nucleic acid amplification detection kits target the highly conserved surface antigen gene or the precore-core genes of the HBV genome [10,12-17] and the HBV DNA controls or standards come from many sources [18-20].

Owing to the lack of a universal HBV DNA standard, identical blood samples yield markedly different HBV DNA levels when tested by different kits [21-23]. These inconsistent results complicate the interpretation of viral load and could influence clinicians+ decisions. Thus, there is an urgent need for a universal and uniform HBV DNA control or standard.

An ideal control or standard should have the following characteristics. First, the standard must be easy to prepare. Second, it must be stabile in storage and during transport. Third, the standard must be noninfectious to humans and not pose a safety issue during manufacture and use. Fourth, the standard should control all steps of the PCR assay, including extraction and amplification. In DNA virus-based tests, the commonly used controls or standards are from 1 of 3 potential sources: plasmid DNA, positive patient specimens, or commercially available viral preparations [11,23,24]. None of these is ideal. Plasmid-derived standards consist of bare, unprotected double-stranded DNA and may become degraded in the clinical specimen before nucleic acid extraction or during storage in the working stock, which could lead to unreliable amplification results. Moreover, plasmid-derived standards could not provide control for the entire PCR assay, because the lysis of viruses during the nucleic acid extraction procedure is not monitored [4,25]. Patient specimens are infectious to humans and, thus, there is some difficulty during manufacture and shipment. Further, viral nucleic acids in patient specimens are degraded during multiple freeze-thaw cycles. Lastly, commercially available viral preparations are heterogeneous or inconsistent from lot to lot. There is a clear need for an improved positive control or standard for DNA quantification that overcomes these limitations [20,26].

There are 3 types of phages that can be used to construct DNA controls or standards: T7 bacteriophage, lambda phage, and filamentous phage [25,27-29]. In the quantitative detection of HBV DNA, the efficiency of nucleic acid purification and amplification of the standards should be identical to the samples. During the early cycles of real-time PCR, primer and probe hybridisation site accessibility is a key aspect in amplification and detection efficiency. The accessibility is primarily determined by the secondary structure of the target nucleic acids [25]. Obviously, single-stranded DNA controls that are derived from filamentous phage cannot mimic HBV double-stranded DNA properties such as charge and secondary structure. Thus, filamentous phage is not an optimal choice for generating DNA controls or standards for the HBV assay. Compared with T7 bacteriophage, lambda phages possess greater packaging efficiency and are easier to obtain [29]. Therefore, we constructed the DNA controls or standards for HBV using lambda phage packaging in vitro.

Lambda phage is a double-stranded DNA phage that can package exogenous DNA into phage particles in vitro. The constructed phage DNA particles are called 'armored DNA'. Armored DNA is produced using mixed bacterial extracts packaging the exogenous DNA in vitro complementation and results in the mature phage particles [28,30]. Armored DNA is DNase-resistant, stable, noninfectious to humans, inexpensive, and easily extracted by conventional methods. Thus, it is an excellent candidate for a positive control in the quantification of a DNA virus.

The first report of lambda phage DNA packaging in vitro was described by Sternberg [31]; it was then optimised by Frackman in 1996 [32]. In the lambda phage, DNA sequences can be packaged and propagated. So far, the largest DNA packaged (425 bp) was prepared using a traditional lambda phage cloning procedure and was used as an internal amplification control by Stocher and Berg [4,20].

In this study, we constructed a double-stranded DNA control or standard that contained a 1,349-bp HBV fragment encompassing the highly conserved surface antigen gene and precore-core genes of the HBV genome using the traditional lambda phage cloning procedure. We show that the obtained double-stranded DNA standard is resistant to DNase I digestion and exhibits improved storage and handling properties. It can likely be used as a universal positive control in quantitative HBV DNA amplification assays.

## Results

### Verification of the Recombinant Plasmid pGEM- HBV_C+S_

**Figure 1:**
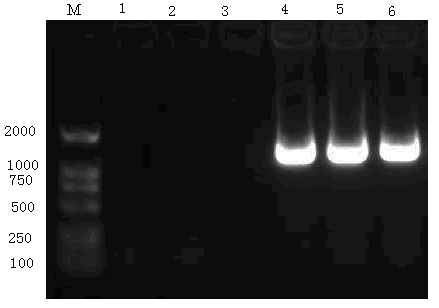
**Verification of the Recombinant pGEM-HBV_C+S _Plasmid**. The PCR amplification products from recombinant pGEM-HBV_c+s _were analysed using ethidium-bromide-stained 1% agarose gel. Lanes 1, 2, and 3: negative control with no template; Lane 4: positive control of recombinant clones; Lane 5 and 6: PCR products of positive recombinant clones.

The PCR products from the recombinant pGEM- HBV_C+S _(using primers 1 and 4) were full length (1,349 bp; Fig. 1). Sequencing also demonstrated that the inserted HBV sequence was 1,349 bp without point mutation. Thus, the recombinant plasmid pGEM- HBV_C+S _was successfully constructed.

### Identification of Armored DNA by Polymerase Chain Reaction and Sequencing

**Figure 2:**
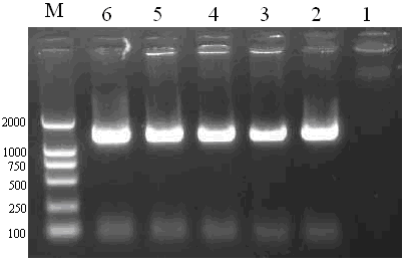
**Identification of Armored DNA by PCR Amplification**. The PCR amplification products of the DNA extracted from lambda phages were analysed using ethidium-bromide stained 1% agarose gel. Lane 1: negative control with no template; Lane 2, 3, 4, 5, and 6: PCR products from positive chimeric phages.

The DNA extracted from lambda phages was amplified with the phage-specific primers gt11-for and gt11-rev. The resulting PCR products were analysed by electrophoresis on agarose gel (1%) containing ethidium bromide (Fig. 2) and sequencing. The result indicated that the sequencing of the PCR products was accurate and the recombinant plasmid gt11-HBV_C+S _were successfully packaged by the phage packaging extracts.

### Durability of Armored DNA

**Figure 3:**
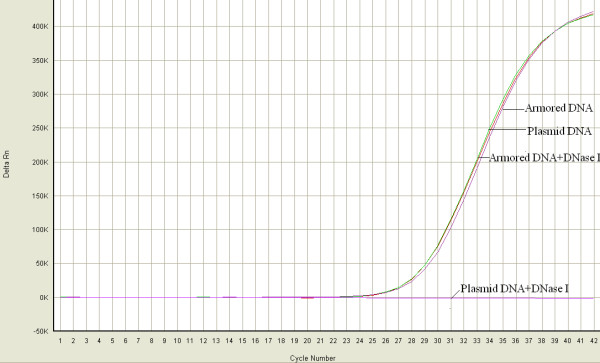
**Durability of Armored DNA**. Equal amounts of armored DNA and plasmid DNA (extracted from the armored DNA) were incubated with DNase I (0.1 units/μl) at 37°C for 60 min. After digestion, the samples were analysed by real-time PCR. The armored DNA was completely resistant to DNase I digestion, whereas plasmid DNA was degraded completely. Armored DNA and plasmid DNA indicate samples without DNase I digestion.

The armored DNA was not degraded by DNase I, whereas the plasmid DNA was completely degraded. This suggested that the generated armored DNA contained intact phage particles that could protect the armored DNA from nuclease degradation (Fig. 3).

### Stability of Armored DNA

**Figure 4:**
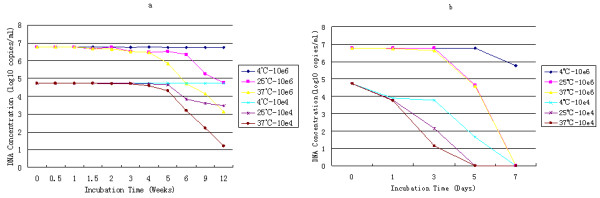
**Stability of the Armored DNA**. (a) Stability of armored DNA in SM buffer. Armored DNA in SM buffer was stable at 4°C for more than 3 months, at 37°C or room temperature was stable for 2 months. The mean for low-copy samples at 4°C was 52,722 copies/ml (4.72 log10; range: 49,239-96,210 copies/ml), and the coefficient of variation was 10.1%. The mean for high-copy samples at 4°C was 5,623,413 copies/ml (6.75 log10; range: 5,510,008-9,575,051 copies/ml), and the coefficient of variation was 6.3%. (b) The stability of armored DNA in plasma. The stability of armored DNA in plasma was poor. The high-copy Armored DNA was not stable for more than 5 days at 4°C, 37°C, or room temperature; the low-copy armored DNA in plasma was not stable for more than 3 days at 4°C, 37°C, or room temperature.

The armored DNA (10^6 ^copies/ml or 10^4 ^copies/ml) in SM buffer at 4°C was stable at least for 3 months; at 37°C or room temperature, it was stable for 2 months. However, the stability of the armored DNA in plasma was poor. The armored DNA (10^6 ^copies/ml) in plasma was not stable for more than 5 days at 4°C, 37°C, or room temperature. At 10^4 ^copies/ml, the armored DNA in plasma was not stable for more than 3 days at 4°C, 37°C or room temperature (Fig. 4).

### Linear Analysis of the Lambda DNA Phage Using a Hepatitis B Virus DNA Fluorescence Quantitative Diagnostic Kit

**Figure 5:**
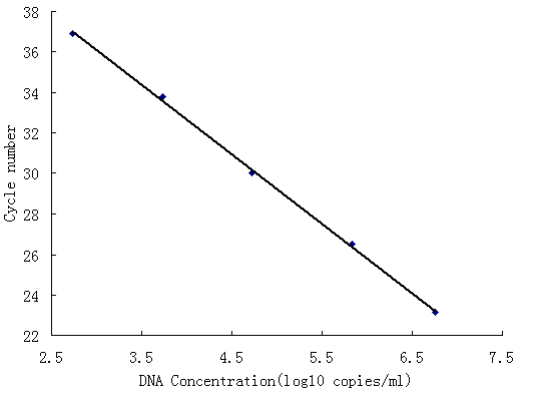
**Linear Analysis of the Lambda DNA Phage Using a HBV DNA fluorescence Quantitative diagnostic kit**. Linearity of the armored DNA was analysed by use of Kehua HBV DNA fluorescence quantitative assay kit. Armored DNA was diluted by 10-fold serial dilutions, and then the samples were quantified using the kit. The samples were tested in triplicate and the mean values were calculated (r^2 ^= 0.99).

To evaluate the performance of armored DNA as a calibrator for HBV DNA assays, we used the National Reference HBV DNA, which was assigned using the HBV international standard (NIBSC 97/746) to calibrate the serially diluted armored DNA. The concentrations of the armored DNA for the 5 dilutions (10^6^, 10^5^, 10^4^, 10^3^, and 10^2^) were 5.85 × 10^6^, 6.81 × 10^5^, 5.23 × 10^4^, 5.45 × 10^3^, and 5.57 × 10^2 ^copies/ml, respectively. A 10-fold dilution of armored DNA produced a linear correlation (r^2 ^= 0.99; Fig. 5). The results demonstrated that armored DNA is capable of performing in HBV DNA fluorescence quantitative assays as a calibration standard for the quantification of HBV DNA.

## Discussion

We constructed an armored DNA particle that can function as a positive control or standard for HBV DNA quantification. During construction, we used the lambda phage gt11 vectors and *Eco*RI restriction enzyme cutting sites in the traditional lambda phage cloning procedure. Because of protection offered by the phage proteins, the obtained armored DNA was resistant to DNase I digestion and exhibited improved storage and handling properties. The armored DNA has reliable amplification in real-time PCR assays. Armored DNA is noninfectious to humans and can be easily extracted by commercial kits; it controls both extraction and amplification of viral DNA from the sample. The constructed armored DNA meets all the requirements of an ideal standard; thus, it may be an excellent positive control or standard for the quantification of DNA viruses.

There is an urgent need for a universal and uniform HBV DNA control or standard for HBV DNA quantification. We aligned all HBV genotypes (A-H) and found that the surface antigen gene and the precore-core gene regions of HBV show the most sequence similarity among the different HBV genotypes [20]. The commercially available HBV nucleic acid detection kits all target the surface antigen gene and the precore-core gene regions of HBV [12-17,33,34]. Therefore, we hypothesised that a chimeric DNA fragment containing all of the conserved sequences of the surface gene and precore-core genes of HBV probably compatible with all HBV nucleic acid amplification detection kits. We made such a chimeric fragment by overlapping extension PCR technology, and then packaged the 1,349-bp fragment into lambda phage. The resulting armored DNA particles were DNase-resistant, stable, noninfectious to humans, easily extracted by conventional methods, and are likely to be compatible with all HBV DNA nucleic acid amplification kits. Thus, the constructed armored DNA control may be used as a universal positive control or standard for HBV DNA quantification.

Although armored DNA has many advantages as a positive control, there is still a problem with its stability. Our experimental data indicated that the armored DNA was not stable for more than 5 days in plasma at room temperature or at 37°C. It is not clear why the lambda phage controls are less stable than the MS2 bacteriophage controls [35], but it may be that lambda is susceptible to plasma proteases. The instability of the armored DNA in plasma does not conflict with its use as a positive control or standard. Owing to its stability in SM buffer, armored DNA diluted in SM buffer may be used as an external positive control or standard for quantifying the samples accurately. At the same time, both nucleic acid extraction and amplification can be well controlled.

## Conclusions

In conclusion, our results demonstrate that the lambda phage packaging system can be used as an excellent expression platform for armored DNA. The obtained armored DNA is DNase resistant and is amplified linearly. It also has improved storage and handling properties and, more importantly, it may be appropriate for all commercial HBV DNA nucleic acid amplification detection kits. Thus, the constructed armored DNA can likely be used as a universal positive control or standard in HBV DNA PCR assays.

## Methods

### Construction of pGEM-HBV_C+S_

An exogenous chimeric sequence (1,349 bp) was constructed from the following sequences: HBV-C (nt1819-2453, 635 bp from the HBV precore-core genes, one of the most conserved regions of HBV), HBV-S (nt125-838, 714 bp from the HBV surface antigen gene, one of the most conserved regions of HBV). The target sequence included the forward primer sites, reverse primer sites, and flanking regions, which are shown in Table 1. We spliced the 2 target DNA sequences together using overlapping extension PCR [36,37]. During the first round of PCR, 2 parts (called fragments C and S) were amplified from template plasmid p57-1 (constructed by our laboratory, GenBank accession no. AY518556) by use of primers 1 and 2 and primers 3 and 4, respectively. Both reactions were performed under the following conditions: denaturing at 94°C for 5 min, followed by a 35-cycle program consisting of 94°C for 30 s, 58°C for 30 s, and 72°C for 1 min, and then 10 min of elongation at 72°C. The PCR products from the first-round amplifications were gel purified and used as templates for the next round of PCR. In the second round of PCR, fragment HBV_C+S _was amplified with primers 1 and 4. The reactions were performed under the following conditions: denaturing at 94°C for 5 min, followed by a 35-cycle program consisting of 94°C for 30 s, 58°C for 30 s, and 72°C for 2 min, and then 10 min of elongation at 72°C. The overlapping extension PCR products were purified and ligated into the pGEM-T Easy vector (Promega Corporation, USA) to generate the recombinant plasmid pGEM-HBV_C+S_. The transformed bacteria were selected by use of blue-white colour identification. The positive constructions were confirmed by PCR and sequencing.

**Table 1:** Primers used in the experiment

Primer name	Primer sequence (5' to 3')
Primer 1	5'-CCG**GAATTC**ACTTTTTCACCTCTGCCTAATCATC-3'
Primer 2	5'-GGTACAGGGTCCCCAATCTTCGATAACTAACATTGGGATTCCCGAGATTG-3'
Primer 3	5'-ATCTCGGGAATCTCAATGTTAGTTATCGAAGATTGGGGACCCTGTACC-3'
Primer 4	5'-CCGGAATTCGGTTTAAATGTATACCCAAAGACAAAAG-3'
gt11-for	5'-CGACTCCTGGAGCCCG-3'
gt11-rev	5'-TGACACCAGACCAACTGGTAATG-3'

Note: The bold sequences are *Eco *RI restriction enzyme sites; the underlined sequences are overlapping nucleotide sequences.

### Packing and Purification of Armored DNA

The HBV_C+S _fragments were excised from plasmids pGEM- HBV_C+S _by use of *Eco*RI restriction enzymes. The HBV_C+S _fragments were purified and inserted into the *Eco*RI cloning site of lambda phage DNA with the use of the lambda gt11/*Eco*RI/CIAP-Treated Vector Kit (Stratagene). A 3-μl portion of this ligation mixture was then used for phage packaging in vitro according to the manufacturer's instruction. The resulting phage particles were transfected into *Escherichia coli *host strain Y1088. After incubation at 37°C for 8 h, single positive plaques were selected by blue-white color identification. Then, the positive phage particles were amplified in *E. coli *strain Y1088 in a separate 10-cm Petri dish according to the manufacturer's instruction. The obtained armored DNA particles were purified by use of the allyl dextran gel chromatography purification system, which was used according to the machine's directions. Finally, the purified armored DNA were stored in SM buffer (100 mmol/L NaCl, 50 mmol/L Tris, 8 mmol/L MgSO_4_, 0.1 g/L gelatin, pH 7.5) at 4°C.

### Identification of Armored DNA Particles by Polymerase Chain Reaction and Sequencing

DNA was extracted from 100 μl of purified armored DNA by using an HBV DNA real-time PCR detection kit (Shanghai KeHua Bio-engineering Co., Ltd.) according to the manufacturer's instruction. The extracted DNA was used as a template for PCR amplification. Primers gt11-for and gt11-rev are sequence-specific primers at both ends of the lambda gt11 vector. PCR was performed in a 50-μl reaction volume, which contained 5 μl of the DNA template, 1× PCR Buffer (TaKaRa, Japan), 1.5 mM MgCl_2_, 300 μM deoxynucleoside triphosphate, 1.5 μM of each primer (gt11-for and gt11-rev), and 1.25 U of PrimerStart Taq DNA polymerase (TaKaRa, Japan). After an initial incubation at 94°C for 5 min, 35 cycles were performed as follows: 94°C for 30 s, 58°C for 30 s, and 72°C for 2 min, followed by a final extension at 72°C for 10 min. The reaction included a negative control with no template, which was tested simultaneously. The PCR products (5 μl) were analysed by electrophoresis on agarose gel (1%) containing ethidium bromide. The PCR products were purified and ligated with the pGEM-T Easy vectors (Promega Corporation, USA) for sequencing.

### Nuclease Resistance of the Lambda DNA Phage

To examine whether the obtained armored DNA was nuclease resistant, we incubated armored DNA with DNase I (0.1 units/μl) at 37°C for 60 min, at the same time, equal amount of plasmid DNA (extracted from the armored DNA) was incubated with DNase I (0.1 units/μl) as the control group. After digestion, the samples were analysed by real-time PCR using a HBV DNA real-time PCR detection kit (Shanghai KeHua Bio-engineering Co., Ltd.).

### Stability of the Armored DNA

The armored DNA was examined for its stability in SM buffer and plasma. Initially, the purified armored DNA was quantified in duplicate using a HBV DNA fluorescence quantitative diagnostic kit (Shanghai Kehua Bio-Engineering Co., Ltd.). The quantified armored DNA was diluted to obtain 10,000 and 1,000,000 copies/ml. For each stability study, a single batch was separated into aliquots of 100 μl for each time point. The samples were then incubated at 4°C, 37°C, or room temperature. The armored DNA samples were quantified at each time by use of a HBV DNA fluorescence quantitative diagnostic kit (Shanghai Kehua). The data were analysed by use of the LightCycler software (Roche).

### Linear Analysis of the Lambda DNA Phage Using a Hepatitis B Virus DNA Fluorescence quantitative diagnostic kit

The purified armored DNA was quantified by use of a HBV DNA fluorescence quantitative diagnostic kit (Shanghai Kehua). We used the National Reference materials as the calibration standards for HBV DNA detection. The National Reference materials were assigned using the WHO international standard for HBV DNA (NIBSC 97/746) assay. The quantified armored DNA was diluted by 10-fold serial dilution to obtain samples containing 10^6 ^to 10^2 ^copies/ml. The samples were then tested in triplicate and the quantification values averaged.

## Competing interests

The authors declare that they have no competing interests.

## Authors' contributions

SM and SZ planned the experimental design and carried out construction of the armored DNA. SM drafted the manuscript. JL conceived the study, participated in its design and coordination, and helped to revise the manuscript. All authors read and approved the final manuscript.
